# Antioxidant Functions of Nitric Oxide Synthase in a Methicillin Sensitive *Staphylococcus aureus*

**DOI:** 10.1155/2013/312146

**Published:** 2013-04-04

**Authors:** Manisha Vaish, Vineet K. Singh

**Affiliations:** Microbiology and Immunology, Kirksville College of Osteopathic Medicine, A.T. Still University of Health Sciences, 800 West Jefferson Street, Kirksville, MO 63501, USA

## Abstract

Nitric oxide and its derivative peroxynitrites are generated by host defense system to control bacterial infection. However certain Gram positive bacteria including *Staphylococcus aureus* possess a gene encoding nitric oxide synthase (SaNOS) in their chromosome. In this study it was determined that under normal growth conditions, expression of *SaNOS* was highest during early exponential phase of the bacterial growth. In oxidative stress studies, deletion of *SaNOS* led to increased susceptibility of the mutant cells compared to wild-type *S. aureus*. While inhibition of *SaNOS* activity by the addition of L-NAME increased sensitivity of the wild-type *S. aureus* to oxidative stress, the addition of a nitric oxide donor, sodium nitroprusside, restored oxidative stress tolerance of the *SaNOS* mutant. The *SaNOS* mutant also showed reduced survival after phagocytosis by PMN cells with respect to wild-type *S. aureus*.

## 1. Introduction

*Staphylococcus aureus* is a Gram-positive bacterial pathogen that colonizes anterior nares and mucosal surfaces in humans and is responsible for causing a wide array of diseases from mild skin infections to life-threatening conditions such as bacteremia, pneumonia, and endocarditis [1–4]. The emerging resistant strains of *S. aureus* exacerbate efforts to control or properly treat staphylococcal infections [5]. 

The host immune system responds to bacterial infections in a concerted manner to eliminate this pathogen. This involves recruitment of polymorphonuclear leukocytes and macrophages to the site of infection and ingestion of invading bacteria. Uptake of bacteria triggers oxygen-dependent and oxygen-independent microbicidal pathways in the phagocytic cells. The oxygen-dependent pathway generates superoxide anion (O_2_^−^) that serves as a precursor for additional reactive oxygen species (ROS) such as hydrogen peroxide (H_2_O_2_), hydroxyl radical, singlet oxygen, hypochlorous acid (HOCl), and peroxynitrite [6–9].

*S. aureus* utilizes various strategies to defend itself against host immune attack. It produces antioxidant enzymes such as superoxide dismutase that converts superoxide anion to H_2_O_2_, catalase that converts H_2_O_2_ to water and oxygen, and alkyl hydroperoxide reductases that detoxify H_2_O_2_, peroxynitrites and hydroperoxides [10, 11]. In addition to their ability to protect from host's oxidants, *S. aureus* infections impose oxidative stress in a host [12]. During infection with a methicillin resistant *S. aureus* strain, host neutrophils respond by an increase in nitric oxide production [12]. Nitric oxide (NO) is a free radical synthesized by nitric oxide synthase. 

Certain Gram-positive bacteria express homologs of nitric oxide synthases (NOS) that have been extensively studied in eukaryotic species. In these species, NOS-derived nitric oxide (NO) is involved in vasodilation, neurotransmission, and host defense [7, 13, 14], but the functions of bacterial NOS are still being defined. Recent genome sequencing has revealed that NOS-like protein exists in many bacteria including *Streptomyces* (StNOS), *Deinococcus* (DrNOS), *Staphylococcus* (SaNOS), and *Bacillus* (BsNOS) species [15]. Bacterial NOS enzymes are homologous with the mammalian NOS, but lack an associated NOS reductase and N-terminal *β*-hairpin hook that binds Zn^2+^, the dihydroxypropyl side chain of H_4_B, and the adjacent subunit of the oxygenase dimer [15–18]. 

It has also been reported that in *Bacillus subtilis*, NO protects bacterial cells from reactive oxygen species [19]. In addition, the *in vivo* survival of *Bacillus anthracis* was dependent on its own NOS activity [20]. NOS activity was also shown to protect from oxidative stress, and deletion of the gene encoding NOS reduced the virulence of a methicillin resistant *S. aureus* [21]. In this study, SaNOS-derived NO was seen to be protective in a methicillin sensitive *S. aureus* from lethal oxidative stress conditions, suggesting its moderate role in stress tolerance.

## 2. Materials and Methods

### 2.1. Bacterial Strains and Growth Conditions

All experiments were carried out using the methicillin sensitive *S. aureus* strain SH1000 (wild-type) [22], its isogenic *SaNOS* deletion mutant, and the mutant complemented with *SaNOS in trans*. Bacterial cultures were grown in tryptic soy broth/agar (TSB/TSA; Becton Dickinson) at 37°C in a shaking (220 rpm) or static incubator. When needed, tetracycline (10 *μ*g mL^−1^) and chloramphenicol (10 *μ*g mL^−1^) were added to the growth medium.

### 2.2. DNA Manipulations and Analysis

Plasmid DNA was isolated using the Qiaprep kit (Qiagen Inc.); chromosomal DNA was isolated using a DNAzol kit (Molecular Research Center) from lysostaphin-treated *S. aureus* cells as per the manufacturer's instructions. All restriction and modification enzymes were purchased from Promega. DNA manipulations were carried out using standard procedures. PCR was performed with the PTC-200 Peltier Thermal Cycler (MJ Research). Oligonucleotide primers were obtained from Sigma Genosys.

### 2.3. Construction of *SaNOS* Mutant

To construct a mutation in the *SaNOS* gene, primers P1 (5′-ACGAATTCTGCTAGCCTTTGTTG-3′) and P2 (5′-GGATCCCAAAATAAACGACCAATGC-3′) were used to amplify an 831 bp DNA fragment using genomic DNA from *S. aureus* strain SH1000 as the template. This amplicon represents *SaNOS* left flanking fragment (starting 207 nt downstream of the *SaNOS* start codon and going upstream). Another set of primers, P3 (5′-GGATCCATTATCTCCAACATTG-3′) and P4 (5′-TCTAGAATCAGCCTGAACGAAAAATCG-3′), was used to amplify an 850 bp DNA fragment representing *SaNOS* right flanking fragment (starting 120 nt upstream of the *SaNOS* stop codon and going downstream). These two fragments were ligated together into vector pTZ18R [23] and a unique *Bam*HI site was engineered between the ligated fragments. To the *Bam*HI site of this fragment (lacking most of the *SaNOS* gene; 750 nt out of a total of 1074 nt of the *SaNOS* gene), a 2.2 kb tetracycline resistance cassette was cloned. The resulting construct was used as a suicidal plasmid to transform *S. aureus* RN4220 cells by electroporation. Transformants were selected on TSA plates containing 10 *μ*g mL^−1^ tetracycline that led to a single crossover event where the mutated *SaNOS* from the plasmid was integrated into the bacterial genome leaving the wild-type *SaNOS* intact. These merodiploids were used to resolve the mutation in the *SaNOS* gene using a phage 80*α* transduction procedure as described previously [24, 25]. Mutation in the *SaNOS* was verified by PCR. For genetic complementation of the *SaNOS* mutant, a 2.4 kb DNA fragment was PCR amplified using primers P1 and P4 and *S. aureus* SH1000 genomic DNA as template. The amplicon represents a fragment starting from 624 nt upstream and spanning 730 nt downstream of the *SaNOS* gene that was cloned into the shuttle plasmid pCU1 [26] and subsequently transferred to the *SaNOS* mutant of *S. aureus* strain SH1000. 

### 2.4. Quantitative Real-Time RT-PCR (qRT-PCR) Assays

qRT-PCR assays were carried out as described [27] using primers P5 (ATGGTGCTAAAATGGCTTGGC) and P6 (GCTTCGTCAGTAACATCTCTTG) to determine optimum expression of *SaNOS* during different stages of *S. aureus* growth in TSB. Bacterial cells were harvested from early- (OD_600_ = 0.6), mid- (OD_600_ = 1.8), late-exponential (OD_600_ = 3.0), and stationary (OD_600_ = 4.2) phase cultures. Total RNA extracted from these cells was used in qRT-PCR assays as described [27].

### 2.5. Determination of Nitric Oxide Synthase Activity

Total protein was extracted from lysostaphin treated *S. aureus* cells grown to OD_600_ = 0.6 as described previously [28]. The NOS activity was determined using NOS activity assay kit (Cayman Chemical Company) and radioactive ^3^H arginine monohydrochloride as substrate (Amersham Biosciences).

### 2.6. Determination of H_**2**_O_**2**_ Susceptibility

For these studies, *S. aureus* cells from early exponential phase cultures OD_600_ = 0.6 were treated with 350 mM H_2_O_2_ for 30 min. The surviving bacteria were enumerated by serial dilution and plating on TSA agar plates. L-arginine serves as a substrate for the nitric oxide synthase in the production of NO. Wild-type *S. aureus* cultures in TSB were added with L-arginine (1 mM final concentration) at OD_600_ = 0.5 and subsequently at OD_600_ = 0.6 were stressed with 350 mM H_2_O_2_ to determine if the addition of L-arginine affected NO production and the oxidative stress tolerance. Additionally, the wild-type *S. aureus* cells were collected from cultures grown to OD_600_ = 0.3 and were resuspended in similar volume of TSB containing 5 mM L-NAME (Tocris Bioscience), an inhibitor of NOS activity. At an OD_600_ = 0.6, these NOS-inhibited cells were stressed with 350 mM H_2_O_2_ for 30 min and the surviving bacteria were counted. To further ascertain the role of nitric oxide in the protection of *S. aureus* cells, the *SaNOS* mutant cells at OD_600_ = 0.5 were treated with 2.5 mM concentration of an NO donor, sodium nitroprusside (SNP) (Sigma). At OD_600_ = 0.6, these SNP-treated cells were stressed with 350 mM H_2_O_2_ for 30 min, and the surviving bacteria were counted.

### 2.7. Phagocytic Killing of *S. aureus SaNOS* Mutant

The promyelocytic HL-60 cells (ATCC) were grown in Iscove's Modified Dulbecco's Medium (IMDM) (ATCC) with 20% fetal bovine serum (Fisher) and were treated with 1.3% DMSO (Fisher) for 5 days to induce their differentiation into neutrophil-like cells [29, 30]. Morphology of differentiated cells was confirmed by Giemsa staining under inverted microscope. The oxidative burst inside neutrophil cells was determined by the reduction of nitroblue tetrazolium. The differentiated neutrophils were used for phagocytic killing using a method described previously [9] with slight modification. In brief, the neutrophils (1 × 10^6^) were added with *S. aureus* cells (2.5 × 10^6^) (MOI 1 : 2.5) in a 24-well plate. The plate was centrifuged at 4000 rpm for 10 min and incubated in a CO_2_ incubator at 37°C for 1 h. The supernatant was gently aspirated and the neutrophils were lysed by the addition of IMDM containing 0.025% Triton X-100. The number of surviving bacteria was enumerated by making serial dilutions and plating of this lysate on TSA plate. 

### 2.8. Statistical Analysis

All results are reported as the mean ± SD of at least three independent experiments. Data were analyzed with Dunnett's Method in one-way analysis of variance or with Student-Newman-Keuls Method in two-way analysis of variance using statistical analysis computer programs (SigmaPlot for Windows, version 12.0, Systat Software, Inc.). Statistical significance was set at *P* < 0.05. 

## 3. Results and Discussion

### 3.1. Construction of *SaNOS* Deletion Mutant in *S. aureus*

**Figure 1:**
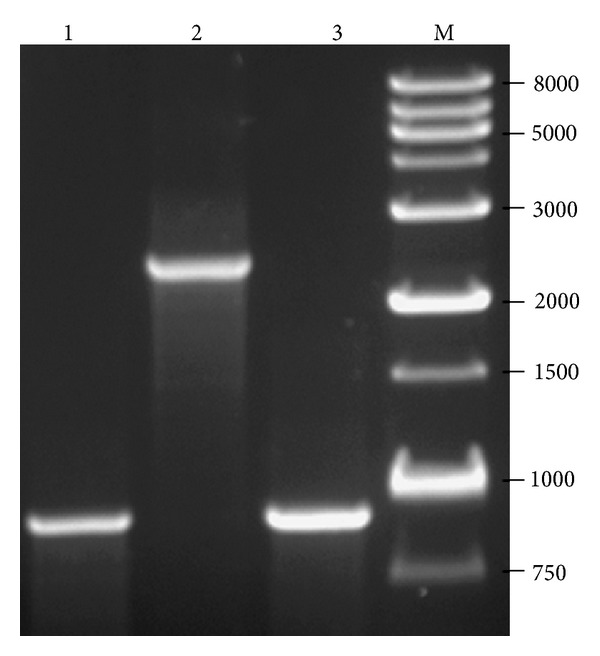
PCR verification of a mutation in the *SaNOS* gene in *S. aureus*. Primers P7 (5′-ATACAGAAGAAGAACTTATTTATGG-3′) and P8 (5′- CACCTCTACTAACTTAATGATGG-3′) were used in the PCR that allowed amplification of a 963 bp product (lane 1) when genomic DNA from wild-type *S. aureus* strain SH1000 was used. These primers amplified a ~2.4 kb fragment when genomic DNA from the *SaNOS* mutant of *S. aureus* strains SH1000 was used as template (lanes 2). Lane 3: PCR product when genomic DNA from the *SaNOS* mutants of *S. aureus* strains SH1000 complemented *in trans* with *SaNOS * was used as template. The larger PCR product is not seen because of complementation with wild-type *SaNOS* gene on a high copy plasmid pCU1. Lane M: DNA ladder.

To investigate the role of the *S. aureus* nitric oxide synthase and NO produced by this enzyme, the *SaNOS* gene was deleted and replaced with a tetracycline cassette by site-directed mutagenesis. The deletion of *SaNOS* gene was confirmed by PCR (Figure 1). 

### 3.2. Expression of *SaNOS* and NOS Enzymatic Activity in *S. aureus*

In qRT-PCR assays, maximum expression of *SaNOS* in strain SH1000 was determined during the early stage of the bacterial growth (Table 1). The expression of *SaNOS* declined dramatically during the late stages of the bacterial growth and was least during the stationary phase (Table 1). A higher bacterial NO production was also noted during the early stages of macrophage infection by *B. anthracis* [19]. The determination of NOS activity, based on the conversion of L-arginine to citrulline, indicated that SaNOS was functional and was able to use L-arginine as the substrate (Table 2). The level of citrulline in the *SaNOS* mutant was similar or below the background level; a reaction mixture that contained only the L-arginine substrate and no protein extract was added to this reaction mixture (Table 2). The complementation of the *SaNOS* mutant with *SaNOS* gene on a high copy plasmid led to a significant increase in the NOS activity in this complemented strain (Table 2). Similar NOS activities in these strains were also verified by measuring the nitrite and nitrate levels using Griess reagent (data not shown). 

### 3.3. Lack of *SaNOS* in *S. aureus* Reduces Its Survival under Oxidative Stress

The impact of the deletion of *SaNOS* was investigated for its growth in TSB. There was no change in the growth of the mutant strain and it was comparable to the growth of the wild-type *S. aureus* (data not shown). Under stress conditions such as salt (1.5 mM NaCl) and pH (6.0 or 8.5), the growth rate of the *SaNOS* was comparable to the growth rate of the wild-type *S. aureus* (data not shown). Also, in the presence of 1.1 mM H_2_O_2_, the growth of the *SaNOS* mutant of *S. aureus* SH1000 was comparable to the wild-type strain (data not shown). However, it has been shown that the priming of the *B. subtilis* cells with nitric oxide for 5 sec leads to a significant increase in their resistance to the exposure of a much higher H_2_O_2_ concentration (370 mM) [19]. 

**Figure 2:**
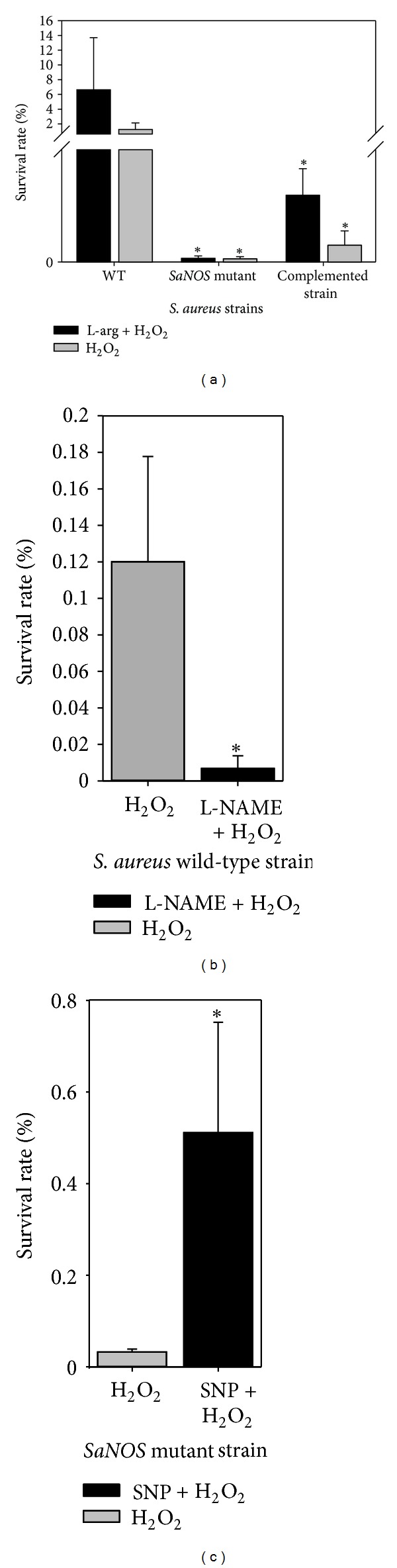
(a) Survival of *S. aureus* SH1000, its isogenic *SaNOS* mutant, and the mutant complemented with *SaNOS* gene *in trans* from a lethal dose (350 mM) of H_2_O_2_ with and without supplementation with 1 mM L-arginine. (b) Survival of wild-type *S. aureus* SH1000 pretreated with 5 mM L-NAME from 350 mM H_2_O_2_. (c) Survival of *SaNOS* mutant of *S. aureus* SH1000 pre-treated with 2.5 mM sodium nitroprusside from 350 mM H_2_O_2_. *Significant at *P* < 0.05.

In qRT-PCR assays, maximum expression of *SaNOS* was determined in the cells from the early exponential phase (OD_600_ = 0.6). Thus, cultures at this density were used in H_2_O_2_ susceptibility assays. When wild-type and the *SaNOS* mutant cells were treated with a lethal dose of 350 mM H_2_O_2_, there were significantly more surviving wild-type bacteria (>1000-fold) compared to the *SaNOS* mutant bacteria under identical experimental conditions (Figure 2(a)). Addition of L-arginine is expected to increase the production of nitric oxide and thus is expected to also increase the resistance of *S. aureus* cells grown in the presence of L-arginine. Addition of L-arginine indeed increased the resistance of the wild-type *S. aureus* cells but caused no increase in the survival of the *SaNOS* mutant (Figure 2(a)). Complementation of *SaNOS* mutant with the *SaNOS* gene on a plasmid partially restored the ability of these bacteria to survive H_2_O_2_ stress when it was grown with or without L-arginine (Figure 2(a)). When the NOS activity was inhibited in the wild-type *S. aureus* by the addition of L-NAME, a competitive inhibitor of the NOS enzymatic activity, it dramatically reduced the bacterial survival (Figure 2(b)) under oxidative stress. In addition, when sodium nitroprusside (an NO donor) was added to the *SaNOS* mutant cells, there was significant increase (>300-fold) in the survival of the mutant bacteria when they were exposed to H_2_O_2_ (Figure 2(c)). These results, collectively, suggest the role of a functional nitric oxide synthase in the protection of *S. aureus* cells from oxidative stress conditions.

### 3.4. Phagocytic Killing of the *SaNOS* Mutant

**Figure 3:**
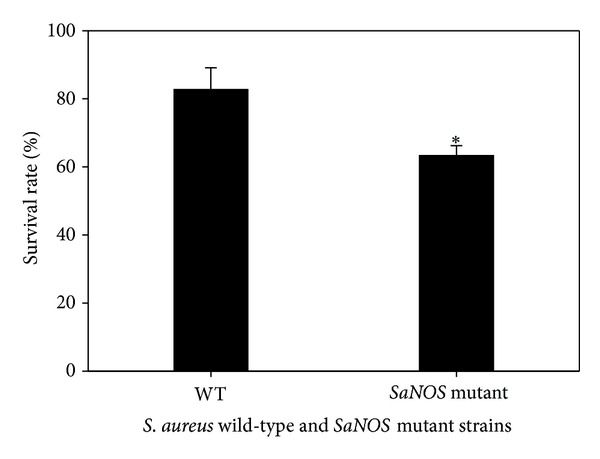
*S. aureus* survival in neutrophil cells. Neutrophil cells were infected (MOI 1 : 2.5) with wild-type *S. aureus* SH1000 and its isogenic *SaNOS* mutant for 1 h at 37°C and then plated on TSA plate. *Significant at *P* < 0.05.

Neutrophils are a critical component of innate immunity and are essential in controlling bacterial infections in a host. Experiments were carried out to determine if the lack of a functional NOS decreased the survival of the *S. aureus* bacteria when it was allowed to interact with neutrophils. In these experiments, the *SaNOS* mutant showed significantly reduced survival compared to the wild-type *S. aureus* (Figure 3). These *SaNOS* mutant bacteria were also used to determine their survival compared to wild-type *S. aureus* in a murine intraperitoneal model as described previously [24, 25]. However, there was no decrease in the survival of the *SaNOS* mutant when compared to the wild-type *S. aureus* bacteria (data not shown). The ability of the *SaNOS* mutant cells to make biofilms was also comparable to the wild-type *S. aureus* cells (data not shown).

In recent years, the presence of NOS has been viewed with great interest for its role in bacterial physiology and virulence. Presence of NOS was determined to be a key factor in the defense of *B. subtilis* and *B. anthracis* from reactive oxygen species generated by the neutrophils and macrophages [19, 20]. It was shown that exposure to nitric oxide enhanced catalase activity in *B. subtilis* [19]. We observed a slight reduction in catalase activity in the *SaNOS* mutant relative to its level in the wild-type *S. aureus* (data not shown). *S. aureus* bacteria are known to produce a very high level of catalase activity. A lower level of superoxide dismutase activity was determined in the *SaNOS* mutant of a methicillin resistant *S. aureus* [21]. The reduced catalase and superoxide dismutase activity levels might be the reasons of the reduced survival of the *SaNOS* mutant under oxidative stress. Lack of the ability of the *S. aureus* cells to produce NO increased the susceptibility to reactive oxygen species and host antimicrobial peptides [21]. The level of the expression of the staphylococcal NOS was induced by exposure to cell wall-active antibiotics and it was also determined to be a factor in conferring resistance to these antibiotics in a methicillin resistant *S. aureus* [21]. Surprisingly, in that study, the lack of a functional NOS increased the resistance of *S. aureus* to aminoglycosides [21].

Studies utilizing a methicillin resistant *S. aureus* showed reduced virulence subsequent to NOS deletion [21]. Infection with the mutant cells resulted in smaller abscess formation compared to the *S. aureus* cell with a functional NOS suggesting its role in staphylococcal virulence [21]. In our studies that utilized a methicillin sensitive *S. aureus*, there was no difference in the survival of the *SaNOS* mutant in a mouse. There was also no appreciable difference in the survival or growth of the *SaNOS* mutant of *S. aureus* SH1000 under mild stress conditions. The difference in the survival was only detected when the *SaNOS* mutant and the wild-type bacteria were exposed to a lethal dose of H_2_O_2_. The reduction in virulence of *S. aureus* subsequent to *SaNOS* deletion in the recent report [21] can be attributed to strain differences (methicillin-resistant versus methicillin-sensitive *S. aureus*) and to a difference in the type of animal model used to study the virulence. These strain differences are significant as host neutrophils respond differently when they are exposed to methicillin-resistant *S. aureus* compared to during infection with methicillin-sensitive *S. aureus* [12]. NO production decreased in neutrophils in mice infected with vancomycin sensitive *S. aureus* and exposed to vancomycin but the decrease in neutrophilic NO production was insignificant when the mice were infected with vancomycin resistant *S. aureus* and exposed to vancomycin [12]. 

During the phagocytic process to control bacterial infections, the respiratory burst generates two very potent toxic substances, H_2_O_2_ and superoxide anions (O_2_^−^). A model has been proposed describing how bacterial NO might be protective from the toxic action of these reactive oxygen species [19, 20]. It is suggested that the O_2_^−^ fails to cross the bacterial cell wall and membrane and limits the production of peroxynitrites inside the bacterial cell from a reaction between bacterial NO and phagocytic O_2_^−^. Although H_2_O_2_ can diffuse inside the bacterial cell, a higher bacterial catalase should degrade it to protect the bacterial cells from any damage. 

Considering the fact that the SaNOS was seen to be significant only during extreme conditions of stress and has a varied role in antibiotic stress tolerance and virulence, more studies need to be carried out to determine the significance of this enzyme in *S. aureus*. 

## Figures and Tables

**Table 1:** Expression of *SaNOS* in *S. aureus* during different phases of growth.

Growth stage	*SaNOS* expression*
Early-exponential	100%
Mid-exponential	19.48%
Late-exponential	10.73%
Stationary	4.90%

*Expression of *SaNOS* is shown relative to its transcript level during early-exponential phase of growth.

**Table 2:** Nitric oxide synthase activity in different *S. aureus* strains.

Strain	NOS activity (%)*
SH1000	3.95 ± 1.61
SH1000Δ*SaNOS *	0
Complemented strain	26.47 ± 3.95

*%Citrulline formed in relation to total L-arginine used in the assay. Citrulline conversion in the mutant strain was below the background level (control reaction with no protein extract). Values represent average of three independent experiments ± standard deviation.
